# High richness of insect herbivory from the early Miocene Hindon Maar crater, Otago, New Zealand

**DOI:** 10.7717/peerj.2985

**Published:** 2017-02-16

**Authors:** Anna Lena Möller, Uwe Kaulfuss, Daphne E. Lee, Torsten Wappler

**Affiliations:** 1 Steinmann Institute for Geology, Mineralogy and Palaeontology, Division Palaeontology, Rheinische Friedrich-Wilhelms Universität Bonn, Bonn, Germany; 2 Department of Geology, University of Otago, Dunedin, New Zealand; 3 Current affiliation: Hessisches Landesmuseum Darmstadt, Darmstadt, Germany

**Keywords:** Early Miocene, Hindon Maar, New Zealand, *Nothofagus*, Plant-insect associations, Southern Hemisphere

## Abstract

Plants and insects are key components of terrestrial ecosystems and insect herbivory is the most important type of interaction in these ecosystems. This study presents the first analysis of associations between plants and insects for the early Miocene Hindon Maar fossil lagerstätte, Otago, New Zealand. A total of 584 fossil angiosperm leaves representing 24 morphotypes were examined to determine the presence or absence of insect damage types. Of these leaves, 73% show signs of insect damage; they comprise 821 occurrences of damage from 87 damage types representing all eight functional feeding groups. In comparison to other fossil localities, the Hindon leaves display a high abundance of insect damage and a high diversity of damage types. Leaves of *Nothofagus*(southern beech), the dominant angiosperm in the fossil assemblage, exhibit a similar leaf damage pattern to leaves from the nearby mid to late Miocene Dunedin Volcano Group sites but display a more diverse spectrum and much higher percentage of herbivory damage than a comparable dataset of leaves from Palaeocene and Eocene sites in the Antarctic Peninsula.

## Introduction

Vascular plants and insects contribute substantially to the Earth’s biodiversity and their interactions constitute a complex and intricate trophic network in terrestrial ecosystems. The origin of these trophic networks can be traced back more than 400 million years (e.g., Labandeira, 2006; Labandeira et al., 2014). As such, the study of fossil plant-arthropod interactions faces many fundamental challenges. The plants have to be identified from often-fragmentary material, and the arthropods that interacted with plants have to be inferred from the traces they left on the plant. Despite the plethora of described terrestrial arthropods globally, fossil evidence for the evolutionary history of these groups in New Zealand is extremely limited: only six insects from Triassic to Eocene were recorded prior to 2007 (e.g., Kaulfuss et al., 2014). Since then, Kaulfuss and colleagues have published a series of papers on the arthropod fauna of the early Miocene laminated, biogenically-varved diatomite succession from Foulden and Hindon Maars, Waipiata Volcanic Field, southern South Island, New Zealand (e.g., Harris, Bannister & Lee, 2007; Kaulfuss, Harris & Lee, 2010; Kaulfuss et al., 2011; Kaulfuss et al., 2014; Kaulfuss et al., 2015; Kaulfuss & Dlussky, 2015; Kaulfuss & Moulds, 2015). Southern New Zealand is also a significant source of early Miocene plant fossils (e.g., Bannister, Conran & Lee, 2012; Bannister, Lee & Raine, 2005; Conran, Bannister & Lee, 2013; Conran et al., 2014; Lee et al., 2010; Lee et al., 2013; Lee et al., 2016; Mildenhall et al., 2014; Nucete, van Konijnenburg-van Cittert & Van Welzen, 2012), including a high proportion of leaves bearing traces of arthropod feeding (Kaulfuss et al., 2014; Reichgelt et al., 2015; Lee et al., 2016).

Deep maar craters, such as Hindon Maar in southern New Zealand are ideal archives for the reconstruction of local palaeoecosystems because the laminated biogenic sediments commonly contain exceptionally preserved fossils. Such records present detailed environmental variables, such as temperature and precipitation changes through timeframes of thousands of years (e.g., Lutz et al., 2010). Although many palaeoecological studies focus on species diversity and distribution, they also provide opportunities to analyse species interactions in great detail within the fossil record (e.g., Wappler et al., 2012; Dunne, Labandeira & Williams, 2014). However, research on changes in food web structures in ecological systems on the basis of palaeontological data is still only at a preliminary stage and underlines our need to understand the impacts of climate change on these systems. In particular, few studies on plant-insect interactions from the Southern Hemisphere have been documented and most are from the Palaeozoic (e.g., Adami-Rodrigues, Iannuzzi & Pinto, 2004; Cariglino & Gutiérrez, 2011; Gallego, Cúneo & Escapa, 2014; McLoughlin, 2011; Pinheiro, Tybusch & Iannuzzi, 2012a; Pinheiro, Iannuzzi & Tybusch, 2012b; Pinheiro et al., 2015; Prevec et al., 2009; more studies are listed in Slater, McLoughlin & Hilton, 2015) and the Mesozoic (Gnaedinger, Adami-Rodrigues & Gallego, 2014; Kellogg & Taylor, 2004; McLoughlin, Martin & Beattie, 2015; Rozefelds & Sobbe, 1987; Scott et al., 2004; Srivastava & Srivastava, 2016; Donovan et al., 2016). Quantitative studies of insect herbivory on Cenozoic Southern Hemisphere dicotyledonous leaf compressions were provided by McDonald (2009) and Reichgelt et al. (2015) who focussed on herbivory patterns on Nothofagaceae from the Palaeocene and Eocene of Antarctica and the Miocene of New Zealand respectively. Previous studies of fossil plant-insect interactions have shown that the herbivory patterns are more complex than expected (e.g., Wappler et al., 2012; Su et al., 2015), and plant-insect interactions typically take place in complex settings of interactions among multiple trophic levels as well as multiple species in each trophic level (Mishra et al., 2015; Wappler et al., 2015).

One of the key issues is the problem of distinguishing the changes in arthropod communities due to changes in plant species composition and due to the reaction of arthropods to climate changes. The use of Neogene plant-arthropod interactions harbours one more important advantage—patterns of damage type diversity and agent host specificity closely resemble those of the present-day (Heie, 1968; Dieguez, Nieves-Aldrey & Barron, 1996; Waggoner & Poteet, 1996; Waggoner, 1999; Erwin & Schick, 2007; Su et al., 2015; Adroit et al., 2016). For these reasons, the Neogene especially represents an extremely interesting and important period in relation to studying the way ecosystems respond to climate fluctuations.

Here, we present the first study on the diversity of insect herbivory on fossil angiosperm leaves from the recently discovered Hindon Maar complex, with emphasis on insect-mediated interactions on plants representing different types of insect feeding guilds in an early Miocene terrestrial ecosystem in New Zealand. The results are interpreted in terms of their palaeoecological implications, in particular for *Nothofagus* which represents an ecologically widespread host taxon in the Southern Hemisphere. *Nothofagus* species are host to a diverse group of herbivores and have been broadly distributed in temperate Southern Hemisphere floras throughout the Cenozoic (e.g., McDonald, 2009; Reichgelt et al., 2015), being the second most common leaf type in the Hindon Maar complex.

## Material and Methods

### Locality and flora

**Figure 1:**
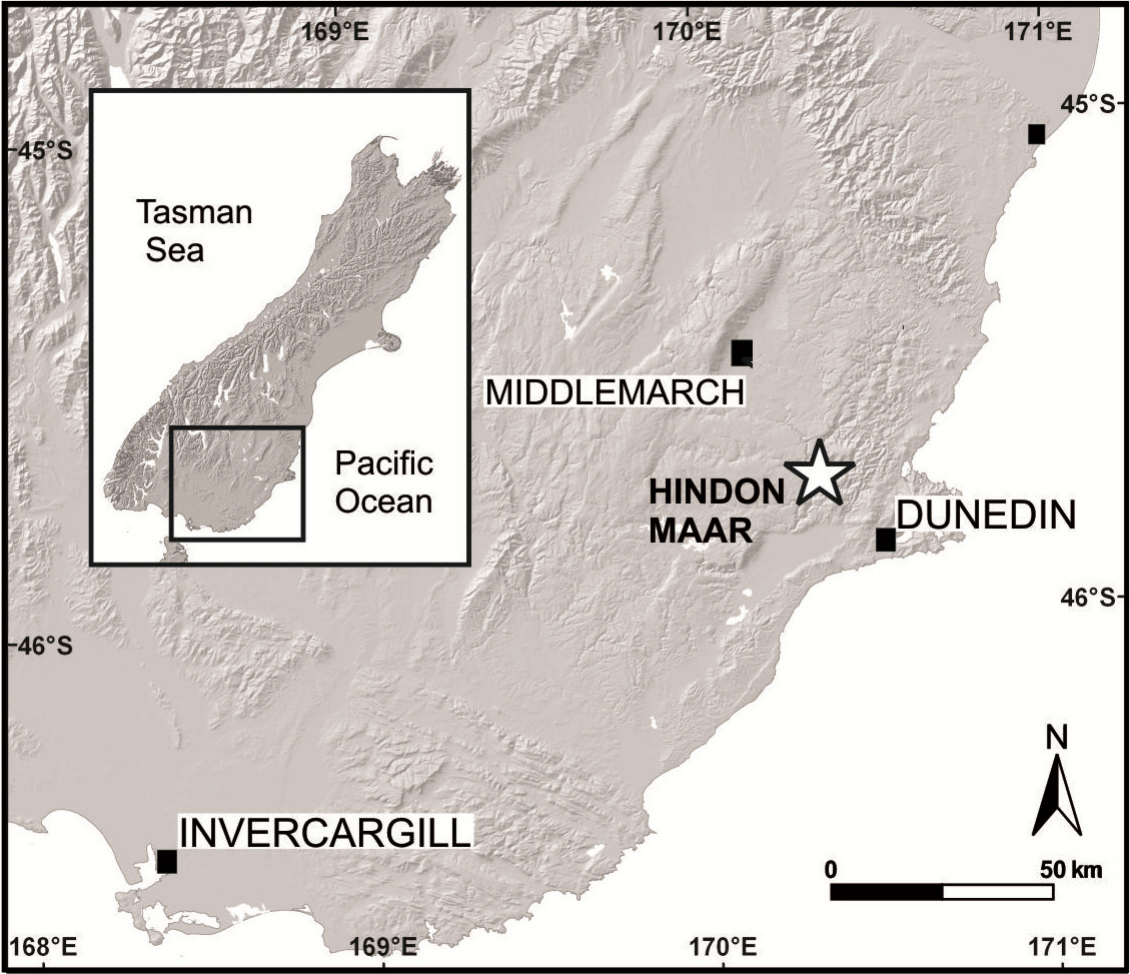
Geographical position of the Hindon Maar locality in southern New Zealand (modified after Kaulfuss & Moulds, 2015).

The Hindon Maar is located at mid-latitudes (palaeolatitude about 45–50°S; Pocknall, 1989) in the Otago region, southern New Zealand, c. 50 km northwest of Dunedin, within the latest Oligocene to mid-Miocene monogenetic Waipiata Volcanic Field (WVF) (45°45.62′S; 170°15.88′E, Fig. 1). The excavation site is situated within a shallow, semi-circular topographic depression (500 × 800 m in diameter) that is infilled by lacustrine mudstones and diatomite, and surrounded by regional metamorphic basement rocks of Jurassic age (Otago Schist) (Kaulfuss & Moulds, 2015). Geological and geophysical data (E Bowie & U Kaulfuss, 2015, unpublished data) indicate that this structure represents the surface expression of a partly eroded maar crater. Leaf fossils studied herein were excavated from laminated, dark brown to black, C_org_-rich, diatomaceous mudstones that were temporarily exposed in a 3 m deep exploration pit in 2014 (I44/f0392 in the New Zealand Fossil Record File). The highly fossiliferous mudstones contain a well-preserved micro- and macroflora, including pollen, spores, leaves, fruits and flowers, as well as numerous insect compression fossils and at least two species of freshwater fish (Kaulfuss & Moulds, 2015). The laminated nature of the sediment, as well as the fossil assemblage present, indicate a calm, lacustrine depositional environment, in which leaves and other plant remains were blown or washed in from forests around the maar lake (Kaulfuss & Moulds, 2015).

The forest occupying the fertile volcanic soil surrounding the Hindon maar lake was a mixed broadleaf rainforest with some podocarps. The most commonly preserved leaves in the lake sediments are species of Nothofagaceae, together with Araliaceae, Lauraceae and Myrtaceae. Cycad foliage is present, and monocots are represented by the liane *Ripogonum* and palm phytoliths and pollen. At least three conifers are represented by foliage and reproductive material (JM Bannister, pers. comm., 2015). All fossil leaves included in the analyses were dicots (non-monocot angiosperms) identifiable to morphotype with at least 50% of the leaf blade intact.

**Figure 2:**
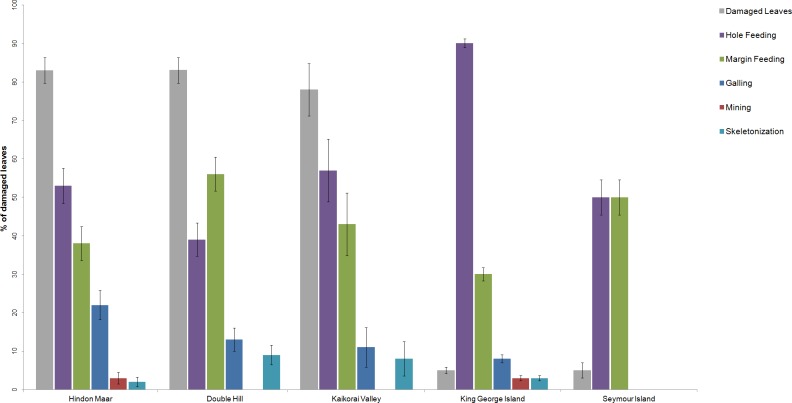
Comparison of the percentages of functional feeding groups and the percentage of damaged leaves present at the Hindon Maar (Miocene), Double Hill (Miocene), Kaikorai Valley (Miocene), King George Island (Palaeocene/Eocene) and Seymour Island (Palaeocene/Eocene). Errors represent binomial confidence intervals. Comparative data from Reichgelt et al. (2015) & McDonald (2009).

Previous work on insect herbivory on *Nothofagus* was made by Russell et al. (2000), McDonald (2006), McDonald (2009), Keble-Williams (2012) and Reichgelt et al. (2015). For a comparison of the herbivory pattern on *Nothofagus* from different localities, data from McDonald (2009), Reichgelt et al. (2015) and the Hindon Maar were used (Fig. 2, Table 1). Fossil material examined by McDonald came from two late Palaeocene and mid-Eocene assemblages in Antarctica (from King George Island and Seymour Island). Reichgelt et al. (2015) studied middle to late Miocene fossil leaves from Double Hill and Kaikorai Valley in the Dunedin Volcanic Group. Surface feeding and piercing and sucking were excluded as they were not analysed in the studies by Reichgelt et al. and McDonald.

**Table 1:** Overview of insect herbivory for *Nothofagus* at Hindon Maar (early Miocene, NZ), Double Hill (middle–late Miocene, NZ), Kaikorai Valley (middle–late Miocene, NZ), King George Island (Palaeocene/Eocene, Antarctica) and Seymour Island (Palaeocene/Eocene, Antarctica). Data from Reichgelt et al. (2015) & McDonald (2009).

Location	# leaves in the census	Damaged leaves (%)	Galling (%)	Hole feeding (%)	Margin feeding (%)	Skeletonization (%)	Mining (%)
Hindon Maar	122	83	22	53	38	2	3
Double Hill	127	83	13	39	56	9	0
Kaikorai Valley	37	78	11	57	43	8	0
King George Island	687	5	8	90	30	3	3
Seymour Island	118	5	0	50	50	0	0

The palynological assemblage of the Hindon Maar deposit suggests an early Miocene age corresponding to the New Zealand stages Otaian to Altonian (21.7–15.9 Ma) (Youngson, 1993), which falls within the radiometrically determined age range of 23–16 Ma for Waipiata volcanism (Coombs et al., 2008).

### Analyses of insect damage

Fossil leaves were examined for signs of insect damage. Insect damage was identified by the presence of reaction tissue (as per Smith, 2008) around missing leaf tissue, and was, in this way, distinguished from leaf tissue removed by mechanical destruction or decay. Herbivory patterns were then categorized based on the widely used damage type (DT) system established by Labandeira et al. (2007). These damage types can be classified into eight functional feeding groups (FFGs), whereby a functional feeding group is an assemblage of DTs caused by the same mode of feeding, such as hole feeding (HF), margin feeding (MF), skeletonization (S), surface feeding (SF), galling (G), mining (M) and piercing and sucking (PS) as well as oviposition (O). Furthermore all damage types have a host specificity value: 1 represents generalized feeding, 2 intermediate conditions and 3 specialized feeding (monophagy) (Wilf & Labandeira, 1999; Labandeira et al., 2007).

Specimens were photographed with a Canon EOS 1100D camera. Photomicrographs were taken with a Canon EOS REBEL T3 camera attached to a Nikon SMZ1000 binocular microscope, and then edited using Adobe Photoshop CS5.1 software.

The fossil material presented here is stored in the Department of Geology, University of Otago, Dunedin.

### Statistical analysis

In order to compare the incidence of different FFGs on leaves from Hindon Maar, rarefaction procedures (rarefaction method without repetition) were applied, using the Vegan Package implement in the R statistics environment (R Development Core Team, 2014). Sample sizes were standardized by selecting a random subset of leaves without replacement and the diversity of damage (all damage types, specialized damage types, galls and mines) for corresponding samples calculated. This process was repeated 5,000 times and the results averaged to obtain a standardized diversity of leaf damage. The standard deviation for the resamples was calculated to provide error bars. The same procedure was used to standardize the overall diversity of leaf damage rarefied to the number of occurrences on all plant taxa or morphotypes based on at least 20 specimens prior to comparison (e.g., Knor et al., 2012; Gunkel & Wappler, 2015). All percentage data, when used, were transformed before analysis as the arcsine, in degrees, of the square root of the proportion equal to the given percentage. Differences between the different plant morphotypes were calculated by the students *t*-test. Pearson‘s chi-square tests were performed in order to investigate the correlation between the number of specimens/percentage of damaged leaves, the number of specimens/damage types and the percentage of damaged leaves/damage types. Correlation between the number of leaves and the percentages of leaves with damage was tested with the correlation coefficient.

## Results

The flora examined includes 584 angiosperm leaves, grouped into 24 leaf morphotypes, except for 118 leaves that were not assignable due to poor and incomplete preservation. Only the six most abundant plant morphotypes are considered in this study. A list of the dicot plant morphotypes found at Hindon Maar, their abundances and associated damage types is given in Table S1. The description of the six most common leaf morphotypes is given in Data S1 using the standard reference (Ellis et al., 1999).

**Figure 3:**
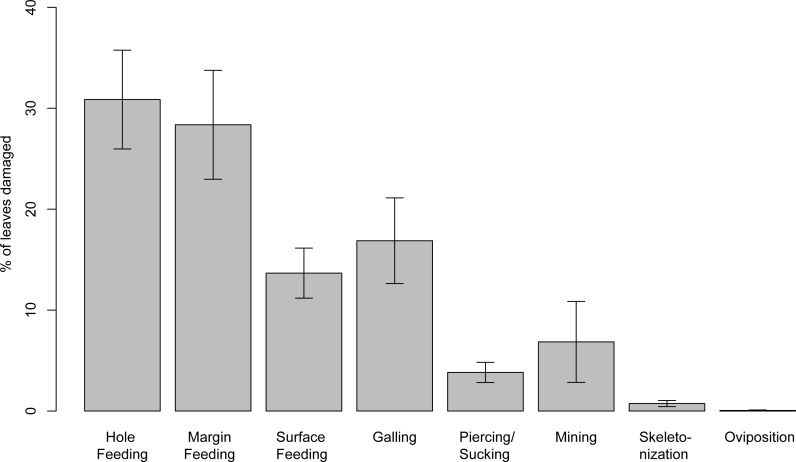
Percentage of recognized functional feeding groups for all leaves examined from the Hindon Maar, early Miocene of New Zealand. Errors represent binomial confidence intervals.

**Figure 4:**
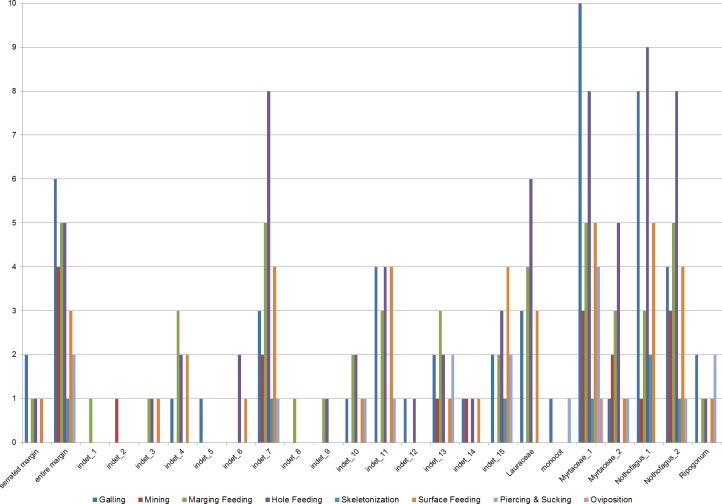
Distribution of functional feeding groups (FFGs) across their leaf morphotypes (Hindon Maar, early Miocene of New Zealand).

Insect damage is found on 73.3% of leaves; this included 821 separate records of damage (including also leaves with two or more DTs), which were categorized into 87 distinct damage types (DTs). On 70% of the leaves of the six most common leaves insect damage was found, including 456 separate records of damage, which were categorized into 60 DTs. The damage types can be categorized into external foliage feeding and internal foliage feeding. The most common functional feeding groups recognized were hole feeding, margin feeding, surface feeding and galling, whereas mining, skeletonization, piercing and sucking, and oviposition were the least abundant (Fig. 3). The distribution of different damage types across their host plants was documented (Fig. 4).

### Composition of leaf damage

#### External foliage feeding

External foliage feeding is among the most difficult of plant-insect interaction types to relate to particular insect taxa since unrelated insect lineages have developed similar feeding strategies and may therefore cause the same types of plant damage (e.g., Labandeira, 1997). Nevertheless, some herbivores can be identified based on specific host plant preferences, prominent patterns based on tissue type and location and homogeneous behaviours of external feeding (Labandeira, 2002b).

#### Hole feeding

**Figure 5:**
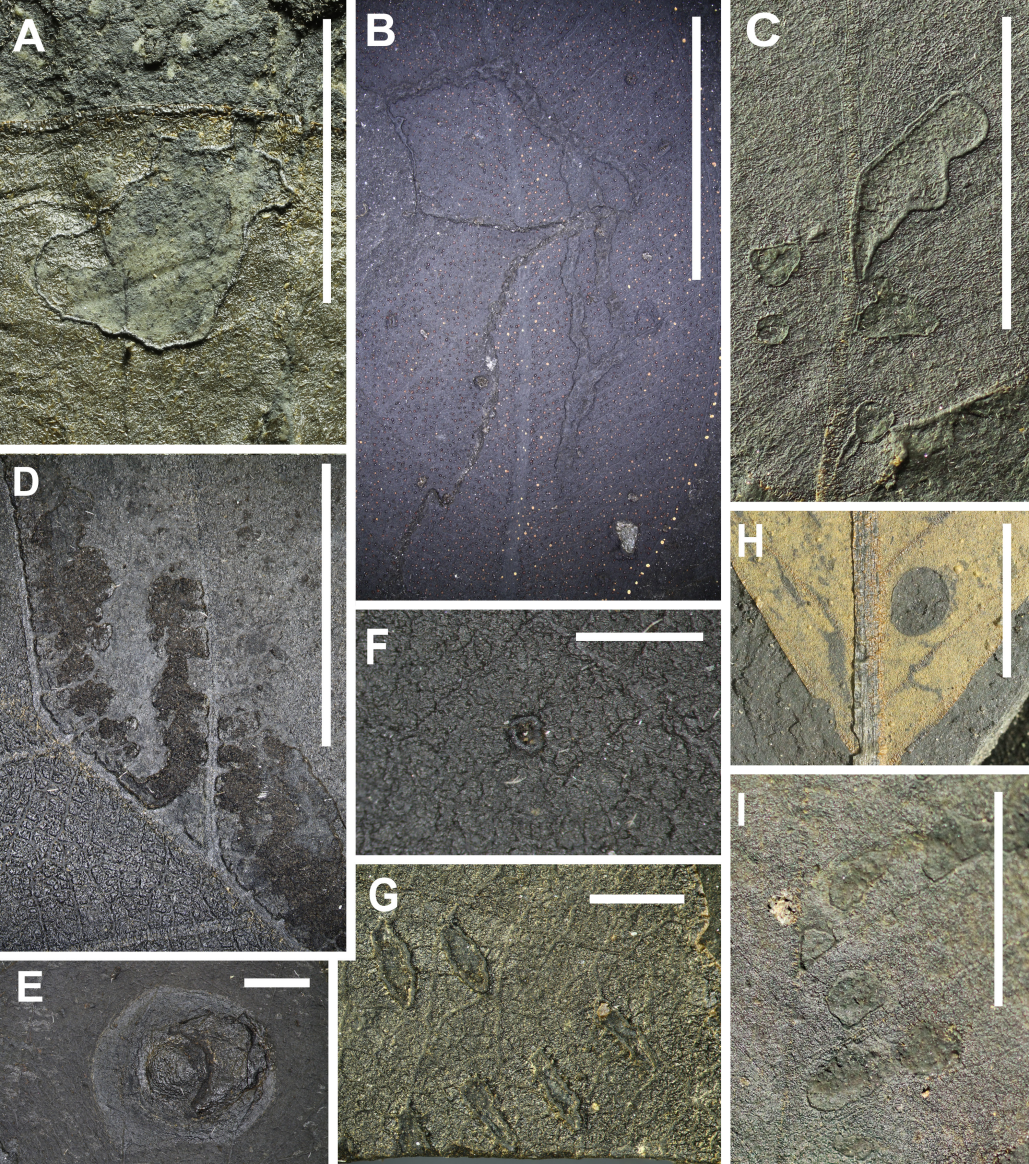
Preservation of leaves and examples of insect damage from Hindon Maar (Miocene, New Zealand). (A) generalized surface abrasion of various sizes and shapes characterized by poorly developed reaction rims (DT29), (B) elongated branched and crisscrossing abrasion of the surface (DT220), (C) surface abrasion characterized by polylobate shape and well developed reaction rim (DT30), medium sized circular perforations (DT2), (D) highly elongate, continuous or patchy skeletonized zone that adjoins and follows one side of the primary and secondary venation (DT61, HS = 3), (E) large, circular scales with a sharp outer lip and a linearly-domed central area (DT158, piercing and sucking), (F) concave styletal puncture into the veins, characterized by an infilling dark, carbonized material and a central depression (DT46, HS = 3, piercing and sucking), (G) lenticular disruptions of surface tissue, arranged in an arcuate row with no preferred orientation on the leaf, typically deployed into subparallel rows (DT54, HS = 3, oviposition), (H) larged-sized, circular perforation (DT4), curvilinear to rectilinear elongated holes that lack parallel sides (DT7), (I) holes lodged at the divergence point of secondary veins from primary veins (DT57, HS = 2), a pattern in which the tissue of three or more intercostal areas is completely removed to varying extent (DT78). All scale: 1 cm (E, F, and G: 1 mm). DT without HS assigned: HS = 1.

[DT1 (93 occurrences), DT2 (88), DT3 (64), DT4 (9), DT5 (13), DT6 (1), DT7 (21), DT8 (3), DT9 (3), DT50 (1), DT57 (10), DT64 (1), DT78 (8), DT113 (2)]. Figs. 5C, 5H and 5I.

Of the 821 damage type occurrences, 317 represent hole feeding, including 14 damage types that vary in diameter (from <1 mm to >5 mm) and shape (circular, comma-shaped or polylobate). Some feeding holes exhibit a broad flange of reaction rim, whereas others are arranged in a linear pattern. Labandeira (2002a) described hole feeding as an excision of interior circular or polygonal pattern. The most frequently observed damage is damage type 1 (DT1), which is described as circular perforations less than 1 mm in diameter, followed by circular perforations 1–5 mm in diameter (DT2) and polylobate perforations of the same size (DT3).

Host plants: serrated margin, entire margin, indet 3, indet 4, indet 6, indet 7, indet 9, indet 10, indet 11, indet 12, indet 13, indet 14, indet 15, Lauraceae, Myrtaceae 1, Myrtaceae 2, *Nothofagus* 1, *Nothofagus* 2, *Ripogonum*.

#### Margin feeding

**Figure 6:**
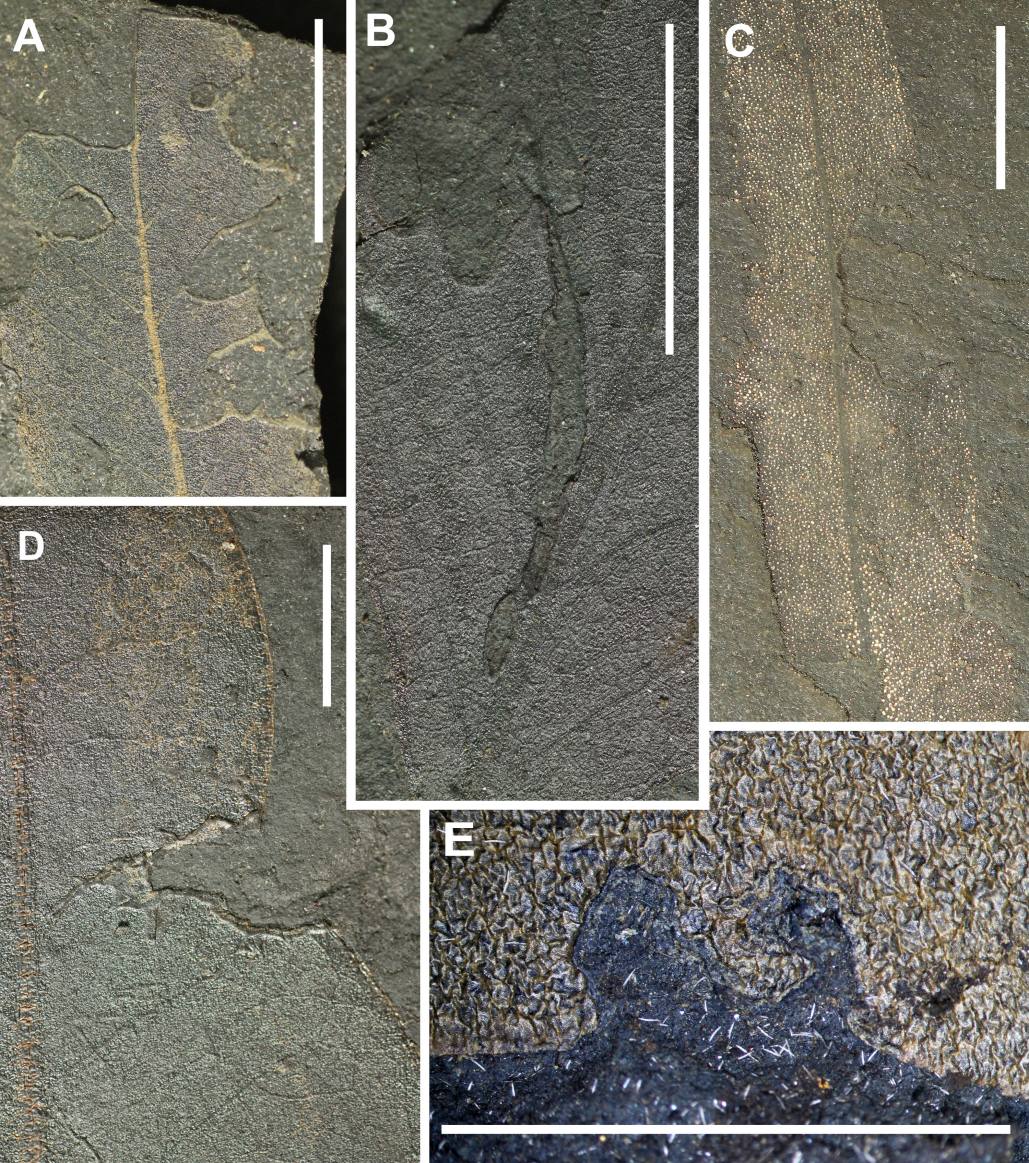
Preservation of leaves and examples of insect margin feeding from Hindon Maar (Miocene, New Zealand). (A) approximately circular excision of a leaf margin for less than 180 degrees of arc (DT12), apex feeding (DT13), excision extending to a primary vein (DT14), (B) highly trenched, narrow margin feeding projecting veinal stringers, extending to the midrib (DT198), (C) Excision of the leaf blade extending to the midvein (DT14), (D) deeply incised excision of the leaf blade expanding inwardly (DT15, HS = 2), (E) near-perfect circular arc of leaf-margin excision, subtending more than 120 degrees of arc (DT81). All scales: 1 cm. DTs without HS: HS = 1.

[DT12 (115 occurrences), DT13 (33), DT14 (14), DT15 (25), DT26 (1), DT81 (2), DT143 (1), DT198 (4)] Fig. 6.

Of the 821 DT occurrences, 195 are margin feeding damage types. They can be assigned to eight types that differ in their position and incision on the leaf. Most common are circular, shallow or deep excisions with less than 180 degrees of arc (DT12). Unlike DT15 and DT26 they show no host specificity. The DT198 differs from that described by Labandeira et al. (2007) in lacking extensive necrotic flaps or adjacent necrotic tissue but having a small reaction rim.

Host plants: serrated margin, entire margin, indet 1, indet, 3, indet 4, indet 7, indet 8, indet 9, indet 10, indet 11, indet 13, indet 15, Lauraceae, Myrtaceae 1, Myrtaceae 2, *Nothofagus* 1, *Nothofagus* 2, *Ripogonum*.

#### Skeletonization

[DT16 (1 occurrence), DT17 (1), DT22 (1), DT61 (4)] Fig. 5D.

Along with oviposition, it is the least common damage observed on leaves from Hindon Maar occurring seven times with four different DTs. Most common is DT61 (four occurrences). This highly elongated, continuous or patchy, unmined skeletonized area adjoins and follows one side of the primary and secondary vein.

Host plants: entire margin, indet 7, indet 15, Myrtaceae 1, *Nothofagus* 1, *Nothofagus* 2.

#### Surface feeding

[DT25 (1 occurrence), DT29 (43), DT30 (26), DT31 (21), DT103 (1), DT130 (1), DT202 (21), DT203 (1), DT207 (2), DT220 (5)] Figs. 5A–5C.

Surface feeding, another type of external foliage feeding, occurred on 122 leaves and 10 different damage types related to surface feeding were identified. The most common types are DT29, DT30 and DT31. The DT29 is a generalized abrasion or window feeding of varied size and shape characterized by poorly developed reaction rims. The DT30 shows surface abrasion or window feeding and is characterized by a polylobate shape and a well-developed reaction rim. The DT31 is characterized by circular to ellipsoidal shapes and a well-developed reaction rim. These damage types are all blotch-like. The DT25, by contrast, is an elongated surface abrasion of constant width but commonly threadlike and branched.

Host plants: serrated margin, entire margin, indet 3, indet 4, indet 6, indet 7, indet 10, indet 11, indet 13, indet 14, indet 15, Lauraceae, Myrtaceae 1, Myrtaceae 2, *Nothofagus* 1, *Nothofagus* 2, *Ripogonum*.

#### Feeding on internal tissues

In contrast to external foliage feeding, feeding on internal tissue takes place inside the leaf where the nutrients are extracted between the upper and lower epidermis. In some cases only the upper or lower layer is attacked, in others both layers (Labandeira, 2002b).

#### Galling

**Figure 7:**
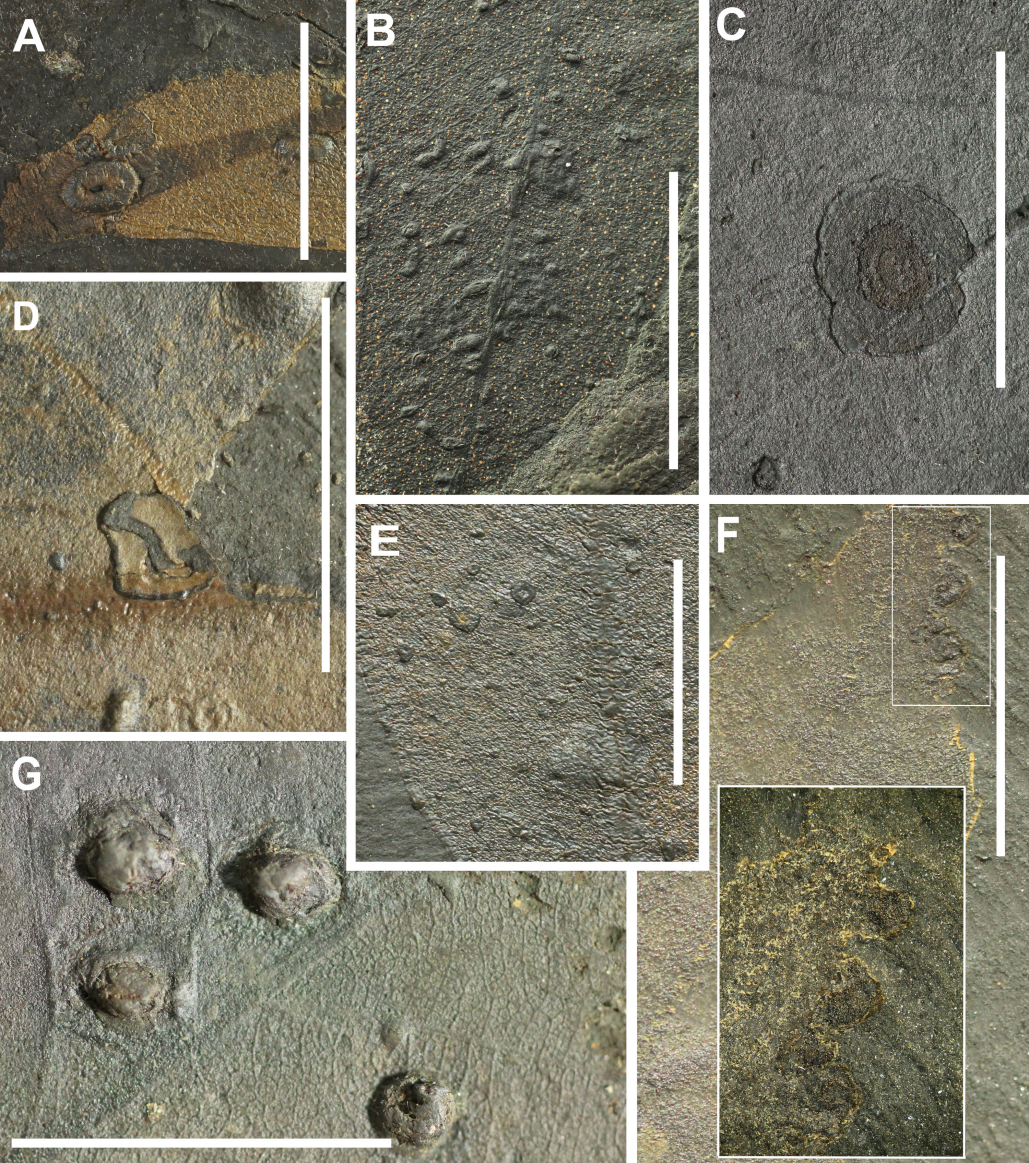
Preservation of leaves and examples of insect galling from Hindon Maar (Miocene, New Zealand). (A) scar of a large gall, occurring in thickened foliar tissue with a central tubular hole (DT194), (B) small galls with diameters up to 1.0 mm characterized by featureless, dark, thickened carbonized material (DT80, HS = 2), (C) Circular blister galls surrounded by a light-hued areola with an eccentric inner chamber (DT218), (D) nondiagnostic, circular to ellipsoidal galls occurring on the interveinal regions (DT32, HS = 2), (E) galls with thin, unhardened central area and surrounded by a thick ring of dense hardened tissue (DT11), (F) equisized circular galls, with a dark outer cover and a single, capacious chamber (DT209), (G) large compound galls enveloped by an outer indurated layer of confluent layer (DT189). All scales = 1 cm. DTs without HS: HS = 1.

[DT11 (9 occurrences), DT32 (11), DT34 (5), DT49 (1), DT52 (1), DT62 (3), DT80 (17), DT117 (2), DT119 (3), DT120 (1), DT145 (2), DT147 (5), DT149 (1), DT153 (1), DT163 (3), DT189 (30), DT 194 (2), DT197 (2), DT209 (1), DT218 (13)] Fig. 7.

In this study, 113 occurrences of galls from 20 gall types were observed. With 30 occurrences, DT189 was most common. This is a large compound gall subdivided by tertiary venation into distinct dark-dyed single-chamber subunits and is encased by an outer indurated layer of confluent tissue (Labandeira et al., 2007). The second most common gall is DT80 with 17 occurrences. These galls are small, hemispherical to rarely more ellipsoidal, ranging in diameter from 0.1 to 1 mm and are built up of featureless, dark, thickened carbonized material. They may occur in clusters and avoid primary and secondary veins. DTs 49, 52, 62, 117, 119, 153 and 163 have high host specificities and are confined to a single plant species or, in some cases, to more than one related species (Labandeira et al., 2007).

Host plants: serrated margin, entire margin, indet 4, indet 5, indet 7, indet 10, indet 11, indet 12, indet 13, indet 14, indet 15, Lauraceae, Myrtaceae 1, Myrtaceae 2, *Nothofagus* 1, *Nothofagus* 2, *Ripogonum*.

#### Mining

**Figure 8:**
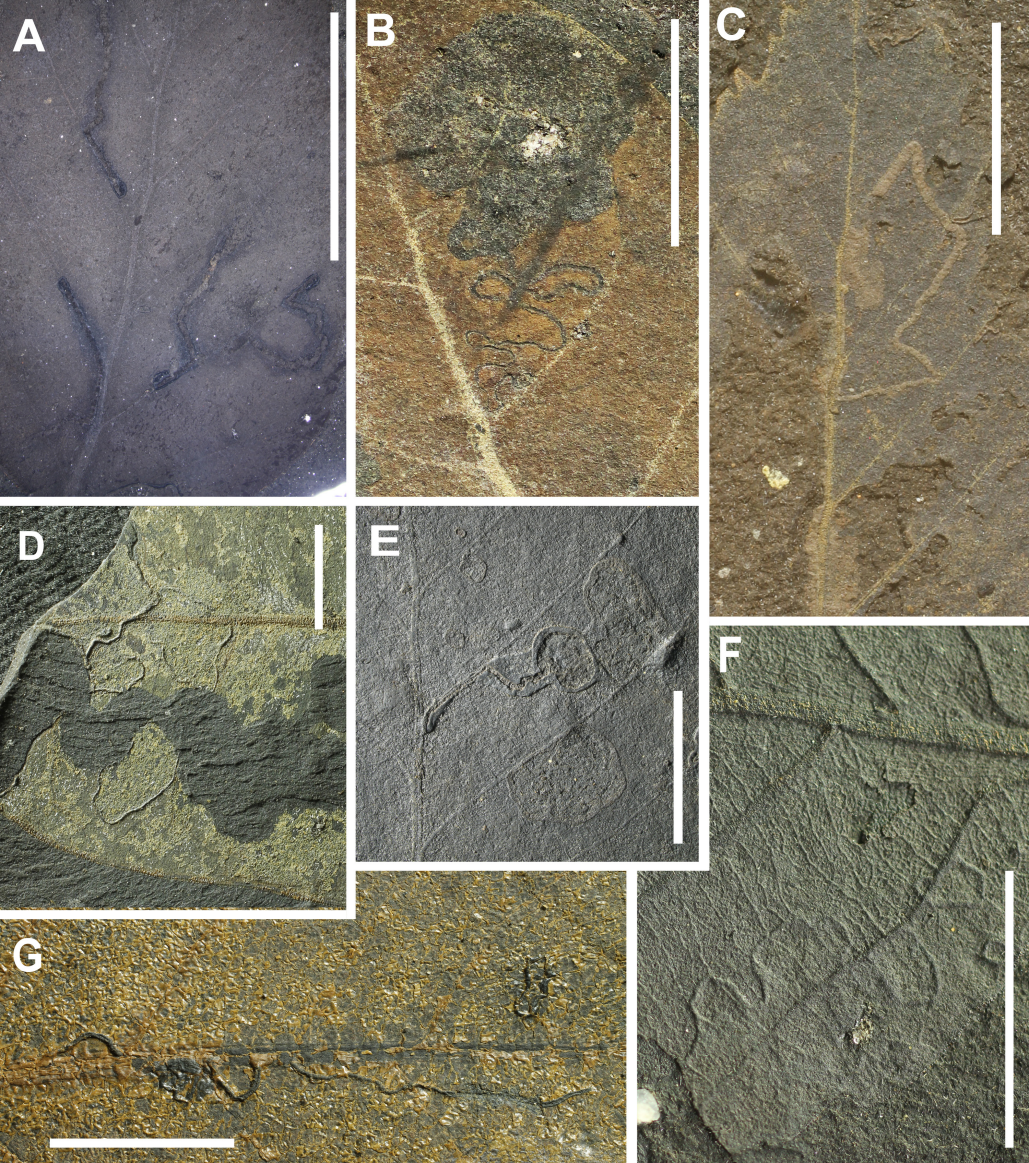
Preservation of leaves and examples of insect mining from Hindon Maar (Miocene, New Zealand). (A) small mines, enlarging in width to ca. 1.0 mm, containing circular frass placed along the medial axis in the last instars (DT210), (B) long, serpentine mine, oviposition site enlarged, frass trail intetiniform with rounded lateral loops, enlarged terminal chamber (DT173, HS = 3), (C) serpentine mine with several folds, crossing secondary and tertiary venation, parallel sided (DT105, HS = 3), (D) highly sinusoidal, crisscrossing mine with a hairline frass trail (DT104), (E) small mine with linear to curved early phase, and a much expanded ovoidal to circular terminal phase (DT176), (F) small, very thin relatively short mine (DT109, HS = 3), G: Serpentine; initially threadlike, then tortuous; undulatory frass packed; margins smooth, increasing width (DT41, HS = 3). All scales = 1 cm. DTs without HS: HS = 1.

[DT35 (1 occurrence), DT36 (1), DT38 (1), DT40 (1), DT41 (1), DT43 (1), DT69 (1), DT90 (1), DT96 (1), DT104 (2), DT105 (2), DT109 (1), DT139 (1), DT171 (1), DT173 (1), DT176 (2), DT185 (1), DT187 (1), DT210 (1)] Fig. 8.

Leaf mining is a highly specialized form of feeding behaviour that gives the insect protection from both predators and dehydration. It consists of tunnelling within plant tissues, especially foliage, by immature insect stages, especially larvae. The leaves from Hindon Maar include 24 occurrences of mining. Among 19 identified mine types there is no dominant type and all have a host specificity of 3 except of DT139 which has a host specificity of 2.

Host plants: entire margin, indet 2, indet 7, indet 13, indet 14, Myrtaceae 1, Myrtaceae 2, *Nothofagus* 1, *Nothofagus* 2.

#### Piercing and sucking

[DT46 (20 occurrences), DT47 (1), DT48 (1), DT128 (2), DT138 (1), DT157 (1), DT158 (4), DT168 (2)] Figs. 5E and 5F.

The studied leaf assemblage hosts 32 occurrences of piercing or sucking marks attributable to eight damage types. The most numerous one is DT46 (20 occurrences), which was described by Labandeira et al. (2007) as concave punctures into the veins, mesophyll or other tissue. It is characterized by an infilling of dark, carbonized material and a central depression. The diameter of these punctures is less than 2 mm and they are distributed in clustered or random patterns. Damage type 46 has a host specificity of 3.

Host plants: entire margin, indet 7, indet 10, indet 11, indet 13, indet 15,, Myrtaceae 1, Myrtaceae 2, *Nothofagus* 2, *Ripogonum*.

#### Oviposition

[DT54 (1 occurrence), DT101 (1)] Fig. 5G.

Possible oviposition sites on leaves are rare in the Hindon Maar flora. There are only two types with one occurrence each. DT54 (on leaf 281) appears as small, ovoid disruptions of the surface tissue surrounded by a rim of prominent scar tissue. They are arranged in consistent rows of up to 20 scars with no preferred orientation on the leaf, typically arranged in multiple subparallel rows that indicate a common pivot point of the ovipositing insect. In this case two rows are identifiable; one of about seven scars, the other of two scars. This damage has a host specificity of 3. The other oviposition damage type is DT101, lenticular to ovoidal scars with prominent reaction rim that occur in an unpatterned and dispersed manner over the leaf surface (Labandeira et al., 2007).

Host plant: Myrtaceae 1.

### Damage frequency on individual leaf morphotypes

**Figure 9:**
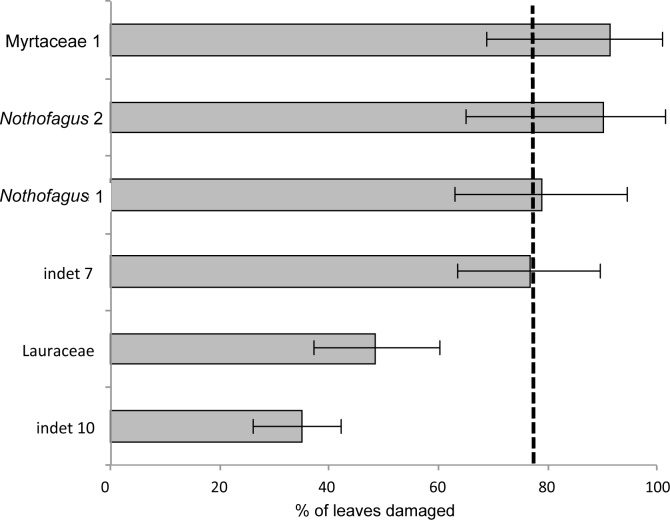
Percentage of damaged leaves for the six most abundant leaf morphotypes from the Hindon Maar (Miocene, New Zealand). Dashed line indicates the mean of damage of the pooled groups (77.8%). Errors represent binomial confidence intervals.

**Figure 10:**
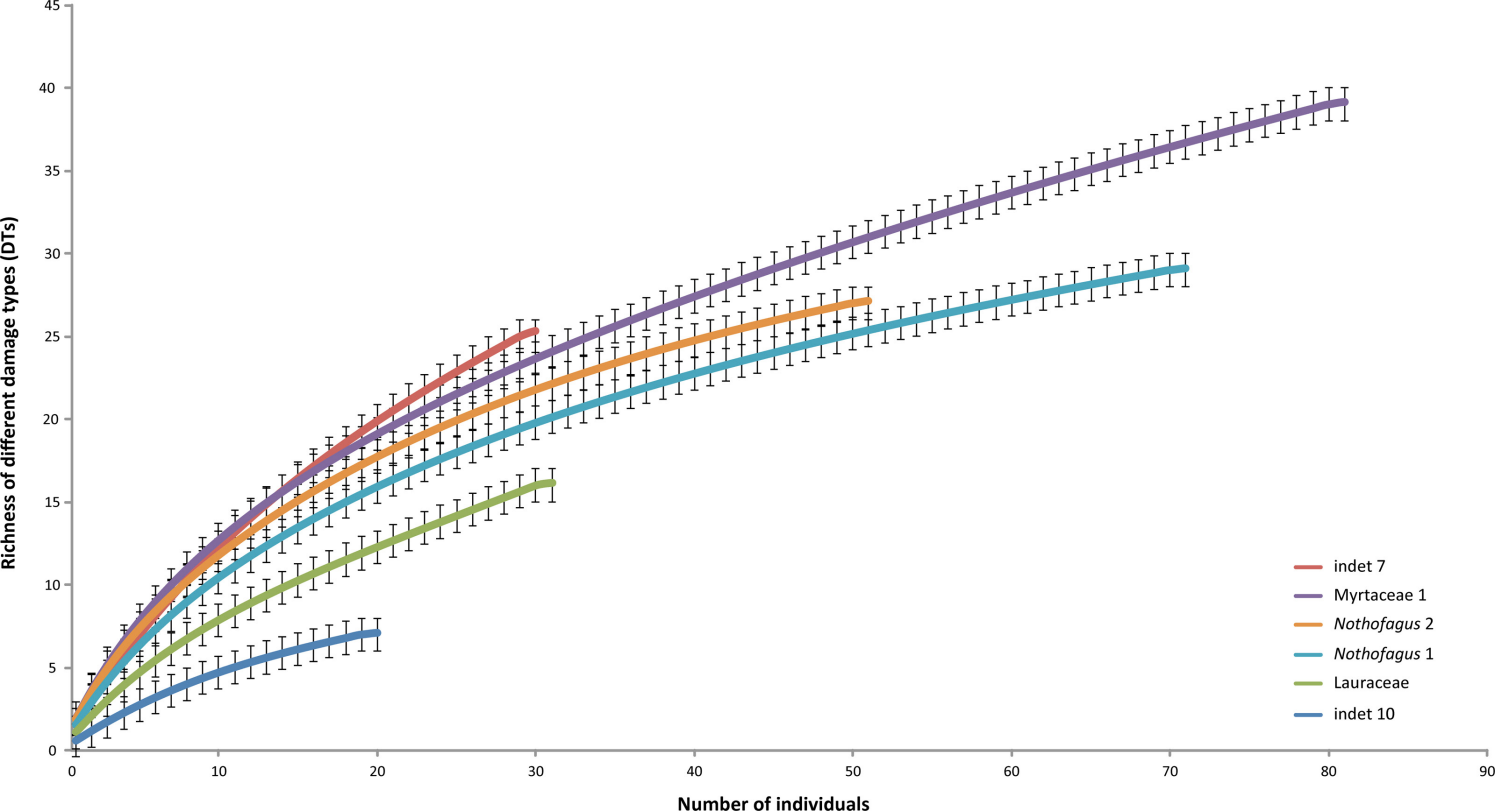
Rarefaction curves of the richness of different damage types represented on the different leaf morphotypes with at least 20 specimens from the Hindon Maar (Miocene, New Zealand). Errors represent binomial confidence intervals.

The most common morphotype is represented by Myrtaceae 1 with 81 specimens (14%) and this is also one of the most frequently consumed species (91.4%) (Fig. 9). Additionally, the richness of 39 different damage types is the highest in the whole assemblage (Fig. 10). The second most common morphotype is *Nothofagus* 1 with 71 specimens (12%) with a proportion of damaged leaves of 78.9%. Despite a considerably lower richness of damage types (29) *Nothofagus 1* is still in second place in this comparison. Third in order of most numerous taxa is *Nothofagus* 2 represented by 51 specimens (9%). It is the second most consumed species (90.2%) with a diversity of damage types of 27. Although Lauraceae (31 specimens) and “indet 7” (30 specimens) both have an abundance of 5%, they differ considerably in their percentage of damaged leaves; Lauraceae 48.4% and “indet 7” 76.7%. “Indet 7” hosts 25 different damage types, Lauraceae only 16. The last of the six most frequent morphotypes is “indet. 10” with 20 specimens (3%). It is the least consumed species (35%) with a diversity of seven damage types. There is no distinct correlation between the number of leaves and the percentage of leaves with insect damage (*r*^2^ = 0.6036, *p* = 0.074).

**Figure 11:**
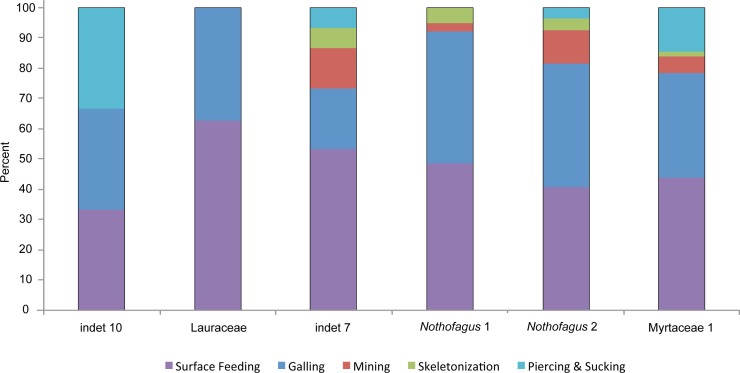
Distribution of functional feeding groups with a high amount of specialized damage types on the six most abundant leaf morphotypes from the Hindon Maar (Miocene, New Zealand).

A closer examination of the five functional feeding groups with a high amount of specialized damage types (piercing and sucking, skeletonization, mining, galling and surface feeding) reveals differences more clearly (Fig. 11). There is no significant correlation between the number of specimens and the number of functional feeding groups (FFG’s). Myrtaceae 1, *Nothofagus* 2 and “indet 7” host five different FFG’s and their abundance is quite different. But, as expected, there is little relationship between the percentages of damaged leaves and the number of FFG’s. All three morphotypes with five FFG’s do have a high level of damaged leaves. Although *Nothofagus* 1 has a slightly higher proportion of damaged leaves than “indet 7”, only four FFG’s are present, which is still relatively high. Notably, Lauraceae contains only two FFG’s (surface feeding and galling) although nearly half of the leaves have herbivory traces. Leaf morphotype “indet 10” has fewer leaves and also a lower percentage of damaged leaves but more functional feeding groups (3) than Lauraceae. Furthermore, it is notable that the galls of “indet. 7” have the smallest number of the FFG’s in comparison to the other five morphotypes; they occurred just half as often. Skeletonization and mining is absent in “indet 10” and Lauraceae. Piercing and sucking is lacking in Lauraceae and *Nothofagus* 1.

The specialized and highly stereotyped kind of damage for the six most frequent leaf morphotypes were compared (Fig. 11). As in all other categories mentioned before, Myrtaceae 1 has the highest value of specialized feeding damage (39.5%) followed by *Nothofagus* 2 (35.3%). Despite exhibiting five FFG’s, “indet 7” does have a comparatively small amount of only 26.7% of specialized damage types. More specialized feeding occurs on *Nothofagus* 1. Lauraceae and “indet 10” show nearly the same percentage of specialized damage (about 15%) though they have different numbers of FFG’s. A clear correlation between the percentage of all damaged leaves and the amount of specialized feeding (*X*^2^ = 14.1021, *df* = 5, *p*-value = 0.01497) is revealed (Figs. 9 and 11).

*Nothofagus* “southern beech” is the dominant fossil plant group in the studied leaves from the Hindon Maar; 122 leaves from at least two species (morphotypes) were found. *Nothofagus* includes 35 living species that are ecologically important trees in many temperate rainforests in Chile, Argentina, Australia and New Zealand, and in subtropical forests in New Caledonia and New Guinea (Dawson, 1966; Heads, 2006). The genus has a rich fossil record, including pollen and numerous macrofossils (Sauquet et al., 2012). The first evidence for all four extant subgenera is in the Upper Cretaceous. All extant species of *Nothofagus* in New Zealand are evergreen although deciduous species were probably present in the Miocene (Pole, 1994).

Extant *Nothofagus* supports about 30 genera of invertebrate herbivores, including both adults and larvae in the orders Lepidoptera, Coleoptera, Phasmatodea and Acari (McQuillan, 1993). Some of these are specialized on *Nothofagus*, potentially reflecting the long history of this genus, which made this specialisation of herbivores possible (McQuillan, 1993). *Nothofagus* has continually responded to many invertebrate species through time and developed a variety of antifeedant chemical defences to become more unpalatable for herbivores (McQuillan, 1993). Coriaceous leaves, hard seed capules, and the presence of phenolic compounds and tannins are indicative of resistance to insect feeding (Russell et al., 2000).

**Figure 12:**
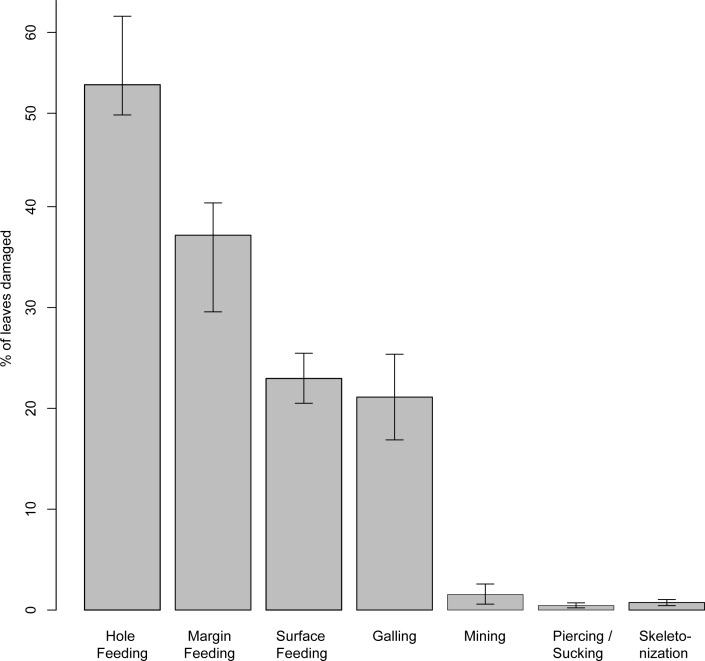
Percentage of each functional feeding group for the summarized *Nothofagus* data from the Hindon Maar (Miocene, New Zealand). Errors represent binomial confidence intervals.

Nevertheless, the two *Nothofagus* morphotypes from the Hindon Maar show a high diversity of herbivory in comparison to the other morphotypes. The percentage of each functional feeding group for the summarized *Nothofagus* data was calculated (Fig. 12).

Based on the results of chi-square tests (*X*^2^ = 189.52, *df* = 20, *p* < 2.2*e* − 16), it can be concluded that the percentage of the functional feeding groups galling, hole feeding and skeletonization differ significantly between each location and through time (from Palaeocene to Miocene), whereas percentages of mining and margin feeding are similar (Table 2). The results of the comparison of the functional feeding groups of the Hindon Maar and the sites of the Dunedin Volcanic Group are more similar than for all studied sites (*X*^2^ = 22.47, *df* = 10, *p* = 0.0128). But differences between the whole data from Hindon Maar and the Dunedin Volcanic Group (Reichgelt et al., 2015) are confirmed by a students *t*-test (*t* = 3.86, *df* = 11, *p* = 0.003). Furthermore, possible differences between (1) Hindon Maar (early Miocene) and both fossil sites of Antarctica (late Palaeocene–mid Eocene; McDonald, 2009) and (2) the Dunedin Volcano Group (middle–late Miocene; Reichgelt et al., 2015) and the two sites of Antarctica were calculated with further statistical *t*-tests. The results show nearly the same conditions for both comparisons with the Antarctic fossil leaves (*t* = 3.15–3.17, *df* = 11, *p* = 0.009). Both New Zealand localities have a much higher percentage of damaged leaves than the two Antarctic sites.

**Table 2:** Differences of the percentages of recognized functional feeding groups between the compared localities. HM, Hindon Maar (early Miocene, NZ); DH, Double Hill (middle–late Miocene, NZ); KV, Kaikorai Valley (middle–late Miocene, NZ); KGI, King George Island (Palaeocene/Eocene, Antarctica); SI, Seymour Island (Palaeocene/Eocene, Antarctica).

Responsible variable	HM (%)	DH (%)	KV (%)	KGI (%)	SI (%)	X^2^	*df*	*p*-value
Galling	22	13	11	8	0	23.59	4	9.6392e–5
Hole feeding	53	39	57	90	50	30.07	4	4.73e–6
Margin feeding	38	56	43	30	50	9.47	4	0.05027
Skeletonization	2	9	8	3	–	13.91	4	0.0076
Mining	3	–	–	3	–	9	4	0.0611

## Discussion

The pattern of insect damage and the intensity of damaged angiosperm leaves from Hindon Maar have enabled a comparison with external variables (e.g., plant diversity and climate) and other plant-insect associational data from the Southern and Northern hemispheres. The presence of all functional feeding groups and an occurrence of 87 damage types indicate a high diversity of fossil insect herbivory on leaves from Hindon Maar. The herbivory types vary in abundance, with hole and margin feeding being the most common and oviposition the least common. The wide diversity of damage types and evidence for high host specificity is apparent as damage frequency depends on the abundance and diversity of insects in the palaeo-area.

Overall, a much higher percentage of leaves with traces of herbivory was identified for the Hindon Maar in comparison to studies from other localities and ages including the Palaeocene from North Dakota (Labandeira, Johnson & Wilf, 2002) and the Menat Pit, France (Wappler et al., 2009); the Palaeocene and early Eocene from Wyoming (Wilf & Labandeira, 1999; Currano et al., 2008; Currano, Labandeira & Wilf, 2010), the Palaeocene and Eocene from Antarctica (McDonald, 2009), the Eocene and Oligocene from Colorado (Smith, 2000), the Eocene from Utah (Wilf et al., 2001) and Eckfeld and Messel, Germany (Wappler et al., 2012), the Miocene from Bilina, Czech Republic (Knor et al., 2012) and the early ‘Tertiary’ from Spitzbergen (Wappler & Denk, 2011) (Table 3). The differences in abundance of leaves with insect damage could be due to changes in diversity of plant-insect associations with time, major ecological crises, environmental perturbations, or preservation bias (Pinheiro, Iannuzzi & Duarte, 2016; Labandeira & Currano, 2013; Wappler et al., 2009).

**Table 3:** Selected publications with information about the locality, age, frequency of herbivory and the number of distinguishable damage types (DTs).

Localities	% of damaged leaves	Number of DTs	Age
Hindon Maar	83	87	Miocene
Double Hill^a^	83	36	Middle Miocene
Kaikorai Valley^a^	78	15	Middle Miocene
King George Island^b^	9,8	54	Late Palaeocene–Eocene
Seymour Island^b^	2,6	19	Late Palaeocene–Eocene
North Dakota^c^	25	51	Palaeocene
Wyoming^d^	29	33	Palaeocene
Wyoming^d^	35	34	Early Eocene
Utah^e^	20	40	Middle Eocene
Colorado^f^	34	n.a.	Eocene
Colorado^f^	23	n.a.	Oligocene
Menat Pit^g^	24–58	39	Middle Palaeocene
Spitzbergen^h^	15–22	35	Early ‘Tertiary’
Messel^i^	21	89	Eocene
Eckfeld^i^	11	68	Eocene
Bilina Mine^j^	18–26	33–54	Lower Miocene

**Notes.**

a Reichgelt et al. (2015).

b McDonald (2009).

c Labandeira, Johnson & Wilf (2002).

d Wilf & Labandeira (1999).

e Wilf et al. (2001).

f Smith (2000).

g Wappler et al. (2009).

h Wappler & Denk (2011).

i Wappler et al. (2012).

j Knor et al. (2012).

For a long time it was suggested that in modern tropical forests insect herbivory is more frequent and diverse than under temperate conditions (e.g., Coley & Barone, 1996). Bale et al. (2002) pointed out that the intensity of biotic interactions is likely to differ among species, and depends on their environments and life-histories as well as their ability to adapt. Similar results were represented by Moles & Ollerton (2016) who analysed different papers on species interactions. The fossil leaves of Hindon Maar show a very high diversity of insect feeding in comparison to floras from Europe and America. An overall similar pattern of insect damage frequency (all DTs) is present in the fossil flora of the Eocene Messel Pit, Germany (Wappler et al., 2012). This pattern was explained by a ca. 2.5-fold increase in atmospheric CO_2_ that overwhelmed evergreen antiherbivore defenses (Wappler et al., 2012) and the richness of these associations exceeds that from other greenhouse peaks, such as the Palaeocene-Eocene Thermal Maximum (PETM) or Early Eocene Climatic Optimum (EECO) (e.g., Wilf & Labandeira, 1999; Currano et al., 2008). Later, Currano, Labandeira & Wilf (2010) suggested that atmospheric CO_2_ levels are associated with damage frequency and damage diversity.

McDonald (2009) suggested that at higher latitudes during the Palaeocene and Eocene the diversity of insect-plant interactions was greater than at mid-latitudes with similar climates. A high diversity of plants stimulates a high diversity of herbivore insects and in tropical zones insects develop up to 14 generations per year, whereas in the temperate zone only one or two generations occur (Archibald et al., 2010). Tropical leaves are generally better defended and less nutritious and this may result in a higher degree of host-plant specialization (Coley & Aide, 1991). However, a high diversity of insect species in tropical communities does not imply higher host specificity than in temperate communities (Novotny et al., 2006).

The Miocene climate of Central Otago was predominantly humid and warm temperate but may have been exposed to periodic drought (Mildenhall, 1989). In the Miocene Manuherikia Group in Central Otago, evidence for frequent fire was documented, suggesting seasonally dry climates, which is supported by the occurrence of Myrtaceae (Mildenhall, 1989; Mildenhall et al., 2014). Myrtaceae is a large tropical to subtropical family in which nearly all species produce ellagic acid, polyphenols against herbivore insects (Haron, Moore & Harborne, 1992). Nevertheless, at Hindon, Myrtaceae display the highest percentage of insect damage among the six most abundant morphotypes and also the highest amount of specialized feeding.

During the Miocene, the climate changed rapidly shortly before the mid–Miocene Climatic Optimum from ever-wet, peat-accumulating to much drier and fire-prone conditions (Pole, 2014), and may be a general phenomenon explaining herbivory intensities. Presumably, the Hindon Maar represents this period and, therefore, combines two types of forests. On the one hand, there are nutrient-rich soils derived from the basaltic tephra rim around the maar. On the other hand, less fertile soils derived from the Otago Schist existed at a greater distance from the maar. In nutrient-poor palaeoenvironments, long-lived leaves are more common, on richer soils deciduous species are dominant (Chabot & Hicks, 1982).

An additional focus was to compare *Nothofagus* leaves from Hindon with other localities and time periods described in McDonald (2006) and Reichgelt et al. (2015). The only other fossil site with a similar high percentage of damaged leaves to Hindon Maar is the *Nothofagus* flora of the Dunedin Volcano Group (Double Hill and Kaikorai Valley) studied by Reichgelt et al. (2015). Using the example of *Nothofagus* it becomes obvious that the fossil leaves from mid-latitude New Zealand have a much higher diversity and occurrence of herbivory than *Nothofagus* leaves of higher latitude fossil sites in Antarctica. One reason could be that the leaves for this study were collected with a focus on insect damage. But, on the other hand, the data from Reichgelt et al. (2015) show a very similar result for the total percentage of damaged leaves. The leaves from Seymour Island were affected by transportation over a long distance and deposition in a high-energy shallow marine environment, which could be one reason for fewer preserved leaves, and, therefore the lower percentage of leaves with insect damage. Nevertheless, the palaeofloral record at all these sites (Table 2) is rich and diverse and further studies may enhance our understanding of climate-related changes of plant-insect interactions at higher latitudes. Despite a similar mean annual temperature (3,5°C–10,4°C) for Seymour and King George Island, the maximum and minimum monthly temperature of the fossil site at Seymour Island was lower (−0.4°C and 16°C on Seymour Island, 3.5°C and 24.3°C on King George Island). Furthermore, the annual precipitation was lower at Seymour Island (570 mm; 1,500 mm at King George Island), so the environmental conditions were more stable (McDonald, 2009). This could have caused lower insect diversity and the apparent lack of galling, mining and skeletonization at this fossil site. Thus, ecological and palaeontological studies indicate that temperature, precipitation, leaf nutrient content, transient increase in floral diversity and change in plant species composition can influence insect herbivory, too (e.g., Currano et al., 2011; Wappler & Grímsson, 2016).

## Conclusion

1. The presence of phytophagous insect activity on fossil angiosperm leaves from the Miocene Hindon Maar has been recorded for the first time. The study shows a very high diversity of insect herbivory (87 DTs) on angiosperm leaves from Hindon Maar (73%).
2. A total of 584 leaves representing 24 leaf morphotypes were examined, which indicate a rich mixed broadleaf rainforest. The most commonly preserved leaves in the lake sediments are species of Nothofagaceae, together with Araliaceae, Lauraceae and Myrtaceae. Monocots are represented by the liane *Ripogonum.* Of these, 118 leaves could not be classified due to poor preservation. The six most abundant morphotypes (at least 20 specimens) represent nearly 50% of all leaves.
3. The herbivory indicates that a wide range of feeding guilds were present, including all eight functional feeding groups; hole feeding, margin feeding, surface feeding, skeletonization, galling, mining, piercing and sucking, and oviposition. For the six most common morphotypes, hole feeding and margin feeding were the most frequent and common damage types.
4. Herbivory on leaves of *Nothofagus* was compared with *Nothofagus* fossil leaves from Antarctica and the Dunedin Volcanic Group. The percentage of damage on *Nothofagus* leaves in the two New Zealand sites is similar but both are much higher than in the Antarctica samples. Both Hindon Maar and the Dunedin Volcanic Group represent isolated, volcanogenic lakes surrounded by probably zonal vegetation which may have had a greater impact on vegetation composition and diversity than differences in climate. Nevertheless, cooler climate conditions could be the main driver to explain the significant difference in diversity and frequency of plant-insect associations in comparison to the Antarctica data.

## Supplemental Information

10.7717/peerj.2985/supp-1 Table S1 List of the plant morphotypes found at Hindon Maar, their abundances and associated damage types

10.7717/peerj.2985/supp-2 Figure S1 Examples of the six most abundant leaf morphotypes from Hindon Maar (A) Nothofagus 1; (B) *Nothofagus* 2; (C) indet. 7; (D) Lauraceae; (E) indet. 10; (F) Myrtaceae 1. Scale bars represent 10 mm.

10.7717/peerj.2985/supp-3 Data S1 raw data, X= Damage Type

## Additional Information and Declarations

### Data Availability

The following information was supplied regarding data availability: The raw data has been supplied as a Supplementary File.
