# Supplementary material for: High richness of insect herbivory from the early Miocene Hindon Maar crater, Otago, New Zealand
Source: PeerJ. 2017 Feb 16;5:e2985. doi: 10.7717/peerj.2985 (PMC5316282; doi:10.7717/peerj.2985)
Supplement: Table S1 [file peerj-05-2985-s001.docx]

| **Species** | **#leaves** | **DMG** | **Spec** | **Gall** | **Mine** | **External** | **MarginF** | **HoleF** | **Skeleton** | **SurfaceF** | **PS** | **Ovi** |
| --- | --- | --- | --- | --- | --- | --- | --- | --- | --- | --- | --- | --- |
| serrated margin | 7 | 57% | 14% | 14% | 0% | 43% | 14% | 14% | 0% | 14% | 0% | 0% |
| entire margin | 45 | 80% | 42% | 13% | 9% | 67% | 27% | 38% | 2% | 18% | 4% | 0% |
| indet_1 | 2 | 50% | 0% | 0% | 0% | 50% | 50% | 0% | 0% | 0% | 0% | 0% |
| indet_2 | 1 | 100% | 100% | 0% | 100% | 0% | 0% | 0% | 0% | 0% | 0% | 0% |
| indet_3 | 2 | 100% | 0% | 0% | 0% | 100% | 50% | 50% | 0% | 50% | 0% | 0% |
| indet_4 | 9 | 67% | 22% | 11% | 0% | 56% | 22% | 44% | 0% | 22% | 0% | 0% |
| indet_5 | 1 | 100% | 0% | 100% | 0% | 0% | 0% | 0% | 0% | 0% | 0% | 0% |
| indet_6 | 7 | 29% | 0% | 0% | 0% | 29% | 0% | 29% | 0% | 14% | 0% | 0% |
| indet_7 | 30 | 77% | 27% | 10% | 7% | 67% | 33% | 40% | 3% | 27% | 3% | 0% |
| indet_8 | 1 | 100% | 0% | 0% | 0% | 100% | 100% | 0% | 0% | 0% | 0% | 0% |
| indet_9 | 1 | 100% | 0% | 0% | 0% | 100% | 100% | 100% | 0% | 0% | 0% | 0% |
| indet_10 | 20 | 35% | 15% | 5% | 0% | 35% | 25% | 20% | 0% | 5% | 5% | 0% |
| indet_11 | 18 | 89% | 56% | 44% | 0% | 78% | 22% | 50% | 0% | 22% | 11% | 0% |
| indet_12 | 5 | 20% | 0% | 20% | 0% | 20% | 0% | 20% | 0% | 0% | 0% | 0% |
| indet_13 | 19 | 42% | 21% | 5% | 5% | 37% | 21% | 16% | 0% | 5% | 11% | 0% |
| indet_14 | 5 | 100% | 20% | 40% | 20% | 40% | 0% | 20% | 0% | 20% | 0% | 0% |
| indet_15 | 16 | 81% | 38% | 31% | 0% | 69% | 31% | 56% | 6% | 19% | 6% | 0% |
| Lauraceae | 31 | 48% | 16% | 10% | 0% | 45% | 26% | 35% | 0% | 16% | 0% | 0% |
| monocot | 7 | 29% | 29% | 14% | 0% | 0% | 0% | 0% | 0% | 0% | 14% | 0% |
| Myrtaceae_1 | 81 | 91% | 40% | 23% | 4% | 80% | 42% | 51% | 1% | 30% | 10% | 1% |
| Myrtaceae_2 | 16 | 69% | 25% | 6% | 13% | 56% | 31% | 44% | 0% | 13% | 13% | 0% |
| *Nothofagus*_1 | 71 | 79% | 31% | 23% | 1% | 69% | 32% | 51% | 3% | 25% | 0% | 0% |
| *Nothofagus*_2 | 51 | 90% | 35% | 22% | 6% | 80% | 47% | 57% | 2% | 22% | 2% | 0% |
| *Ripogonum* | 16 | 25% | 19% | 13% | 0% | 13% | 6% | 6% | 0% | 6% | 13% | 0% |
| Total | 584 | 73% | 31% | 18% | 4% | 62% | 29% | 41% | 1% | 20% | 5% | 0% |

| **Species** | **DTs** | **SpecDTs** | **Gall** | **Mine** | **ExtDTs** | **MarginF** | **HoleF** | **Skeleton** | **SurfaceF** | **PS** | **Ovi** | **#FFGs** |
| --- | --- | --- | --- | --- | --- | --- | --- | --- | --- | --- | --- | --- |
| serrated margin | 5 | 2 | 2 | 0 | 3 | 1 | 1 | 0 | 1 | 0 | 0 | 4 |
| entire margin | 26 | 15 | 6 | 4 | 13 | 5 | 5 | 1 | 3 | 2 | 0 | 7 |
| indet_1 | 1 | 0 | 0 | 0 | 1 | 1 | 0 | 0 | 0 | 0 | 0 | 1 |
| indet_2 | 1 | 1 | 0 | 1 | 0 | 0 | 0 | 0 | 0 | 0 | 0 | 1 |
| indet_3 | 3 | 0 | 0 | 0 | 3 | 1 | 1 | 0 | 1 | 0 | 0 | 3 |
| indet_4 | 8 | 2 | 1 | 0 | 7 | 3 | 2 | 0 | 2 | 0 | 0 | 4 |
| indet_5 | 1 | 0 | 1 | 0 | 0 | 0 | 0 | 0 | 0 | 0 | 0 | 1 |
| indet_6 | 3 | 0 | 0 | 0 | 3 | 0 | 2 | 0 | 1 | 0 | 0 | 2 |
| indet_7 | 25 | 8 | 3 | 2 | 17 | 5 | 8 | 1 | 4 | 1 | 0 | 7 |
| indet_8 | 1 | 0 | 0 | 0 | 1 | 1 | 0 | 0 | 0 | 0 | 0 | 1 |
| indet_9 | 2 | 0 | 0 | 0 | 2 | 1 | 1 | 0 | 0 | 0 | 0 | 1 |
| indet_10 | 7 | 3 | 1 | 0 | 5 | 2 | 2 | 0 | 1 | 1 | 0 | 5 |
| indet_11 | 17 | 5 | 4 | 0 | 11 | 3 | 4 | 0 | 4 | 1 | 0 | 5 |
| indet_12 | 2 | 0 | 1 | 0 | 1 | 0 | 1 | 0 | 0 | 0 | 0 | 2 |
| indet_13 | 11 | 5 | 2 | 1 | 6 | 3 | 2 | 0 | 1 | 2 | 0 | 6 |
| indet_14 | 4 | 1 | 1 | 1 | 2 | 0 | 1 | 0 | 1 | 0 | 0 | 4 |
| indet_15 | 14 | 5 | 2 | 0 | 9 | 2 | 3 | 1 | 4 | 2 | 0 | 6 |
| Lauraceae | 16 | 5 | 3 | 0 | 13 | 4 | 6 | 0 | 3 | 0 | 0 | 4 |
| monocot | 2 | 2 | 1 | 0 | 0 | 0 | 0 | 0 | 0 | 1 | 0 | 2 |
| Myrtaceae_1 | 39 | 19 | 10 | 3 | 18 | 5 | 8 | 1 | 5 | 4 | 1 | 7 |
| Myrtaceae_2 | 13 | 4 | 1 | 2 | 9 | 3 | 5 | 0 | 1 | 1 | 0 | 6 |
| *Nothofagus*_1 | 29 | 13 | 8 | 1 | 17 | 3 | 9 | 2 | 5 | 0 | 0 | 6 |
| *Nothofagus*_2 | 27 | 10 | 4 | 3 | 17 | 5 | 8 | 1 | 4 | 1 | 0 | 7 |
| *Ripogonum* | 7 | 4 | 2 | 0 | 3 | 1 | 1 | 0 | 1 | 2 | 0 | 5 |
| Total | 87 | 54 | 20 | 19 | 32 | 8 | 14 | 4 | 10 | 8 | 2 | 7 |

| **Species** | **DTOAll** | **DTOSpec** | **DTOGall** | **DTOMine** | **DTOExternal** | **DTOMF** | **DTOHF** | **DTOSK** | **DTOSF** | **DTOPS** | **DTOOvi** |
| --- | --- | --- | --- | --- | --- | --- | --- | --- | --- | --- | --- |
| serrated margin | 5 | 2 | 2 | 0 | 3 | 1 | 1 | 0 | 1 | 0 | 0 |
| entire margin | 58 | 19 | 6 | 4 | 45 | 14 | 23 | 1 | 8 | 2 | 0 |
| indet_1 | 1 | 0 | 0 | 0 | 1 | 1 | 0 | 0 | 0 | 0 | 0 |
| indet_2 | 1 | 1 | 0 | 1 | 0 | 0 | 0 | 0 | 0 | 0 | 0 |
| indet_3 | 3 | 0 | 0 | 0 | 3 | 1 | 1 | 0 | 1 | 0 | 0 |
| indet_4 | 11 | 2 | 1 | 0 | 10 | 3 | 5 | 0 | 2 | 0 | 0 |
| indet_5 | 1 | 0 | 1 | 0 | 0 | 0 | 0 | 0 | 0 | 0 | 0 |
| indet_6 | 3 | 0 | 0 | 0 | 3 | 0 | 2 | 0 | 1 | 0 | 0 |
| indet_7 | 46 | 10 | 4 | 2 | 37 | 11 | 17 | 1 | 9 | 1 | 0 |
| indet_8 | 1 | 0 | 0 | 0 | 1 | 1 | 0 | 0 | 0 | 0 | 0 |
| indet_9 | 2 | 0 | 0 | 0 | 2 | 1 | 1 | 0 | 0 | 0 | 0 |
| indet_10 | 12 | 4 | 1 | 0 | 10 | 5 | 4 | 0 | 1 | 1 | 0 |
| indet_11 | 29 | 10 | 8 | 0 | 18 | 4 | 10 | 0 | 4 | 2 | 0 |
| indet_12 | 2 | 0 | 1 | 0 | 1 | 0 | 1 | 0 | 0 | 0 | 0 |
| indet_13 | 14 | 5 | 2 | 1 | 9 | 4 | 4 | 0 | 1 | 2 | 0 |
| indet_14 | 5 | 1 | 2 | 1 | 2 | 0 | 1 | 0 | 1 | 0 | 0 |
| indet_15 | 29 | 8 | 5 | 0 | 21 | 6 | 11 | 1 | 4 | 2 | 0 |
| Lauraceae | 34 | 5 | 3 | 0 | 31 | 10 | 16 | 0 | 5 | 0 | 0 |
| monocot | 2 | 2 | 1 | 0 | 0 | 0 | 0 | 0 | 0 | 1 | 0 |
| Myrtaceae_1 | 157 | 37 | 21 | 3 | 118 | 40 | 51 | 1 | 27 | 9 | 1 |
| Myrtaceae_2 | 20 | 5 | 1 | 2 | 15 | 5 | 8 | 0 | 2 | 2 | 0 |
| *Nothofagus*_1 | 110 | 24 | 17 | 1 | 87 | 24 | 45 | 2 | 18 | 0 | 0 |
| *Nothofagus*_2 | 97 | 20 | 11 | 3 | 80 | 29 | 40 | 1 | 11 | 1 | 0 |
| *Ripogonum* | 7 | 4 | 2 | 0 | 3 | 1 | 1 | 0 | 1 | 2 | 0 |
| Total | 821 | 201 | 113 | 22 | 634 | 195 | 317 | 7 | 122 | 32 | 2 |

| **Species** | **DTs present** |
| --- | --- |
| serrated margin | 4;13;31;80;209 |
| entire margin | 1;2;3;4;6;12;13;14;15;29;30;31;32;61;80;81;104;109;128;158;163;176;185;189;194;218 |
| indet_1 | 12 |
| indet_2 | 104 |
| indet_3 | 2;12;31 |
| indet_4 | 2;7;12;13;15;29;30;163 |
| indet_5 | 149 |
| indet_6 | 2;7;29 |
| indet_7 | 1;2;3;5;7;9;12;13;14;15;17;29;30;31;34;35;40;46;58;62;78;113;143;189;202 |
| indet_8 | 12 |
| indet_9 | 3;12 |
| indet_10 | 1;3;12;15;29;32;46 |
| indet_11 | 1;2;3;7;12;13;14;30;31;32;34;46;58;80;117;202;220 |
| indet_12 | 1;147 |
| indet_13 | 2;4;12;13;14;30;36;46;153;158;194 |
| indet_14 | 2;11;31;139 |
| indet_15 | 1;2;7;12;13;29;30;31;46;48;61;189;202;218 |
| Lauraceae | 1;2;3;7;8;12;13;14;15;29;30;62;64;147;218;220 |
| monocot | 46;80 |
| Myrtaceae_1 | 1;2;3;5;7;8;11;12;13;15;22;29;30;31;32;34;43;46;47;57;58;78;80;81;96;101;103;106;117;119;120;147;157;168;176;189;198;202;218 |
| Myrtaceae_2 | 1;2;3;4;5;12;13;14;46;90;119;173;220 |
| *Nothofagus*_1 | 1;2;3;7;8;9;11;12;13;15;16;29;30;31;32;50;57;58;61;62;78;80;105;145;163;189;202;207;218 |
| *Nothofagus*_2 | 1;2;3;4;5;7;11;12;13;14;15;29;30;31;41;46;57;58;61;78;80;187;189;198;202;210;218 |
| *Ripogonum* | 3;12;30;46;49;138;197 |
| Total | 1;2;3;4;5;6;7;8;9;11;12;13;14;15;16;17;22;25;26;29;30;31;32;34;35;36;38;40;41;43;46;47;48;49;50;52;54;57;58;61;62;64;69;78;80;81;90;96;101;103;104;105;106;109;113;117;119;120;128;130;138;139;143;145;147;149;153;157;158;163;168;171;173;176;185;187;189;194;197;198;202;203;207;209;210;218;220 |
