# Supplementary figures and images for: High richness of insect herbivory from the early Miocene Hindon Maar crater, Otago, New Zealand
Source: PeerJ. 2017 Feb 16;5:e2985. doi: 10.7717/peerj.2985 (PMC5316282; doi:10.7717/peerj.2985)

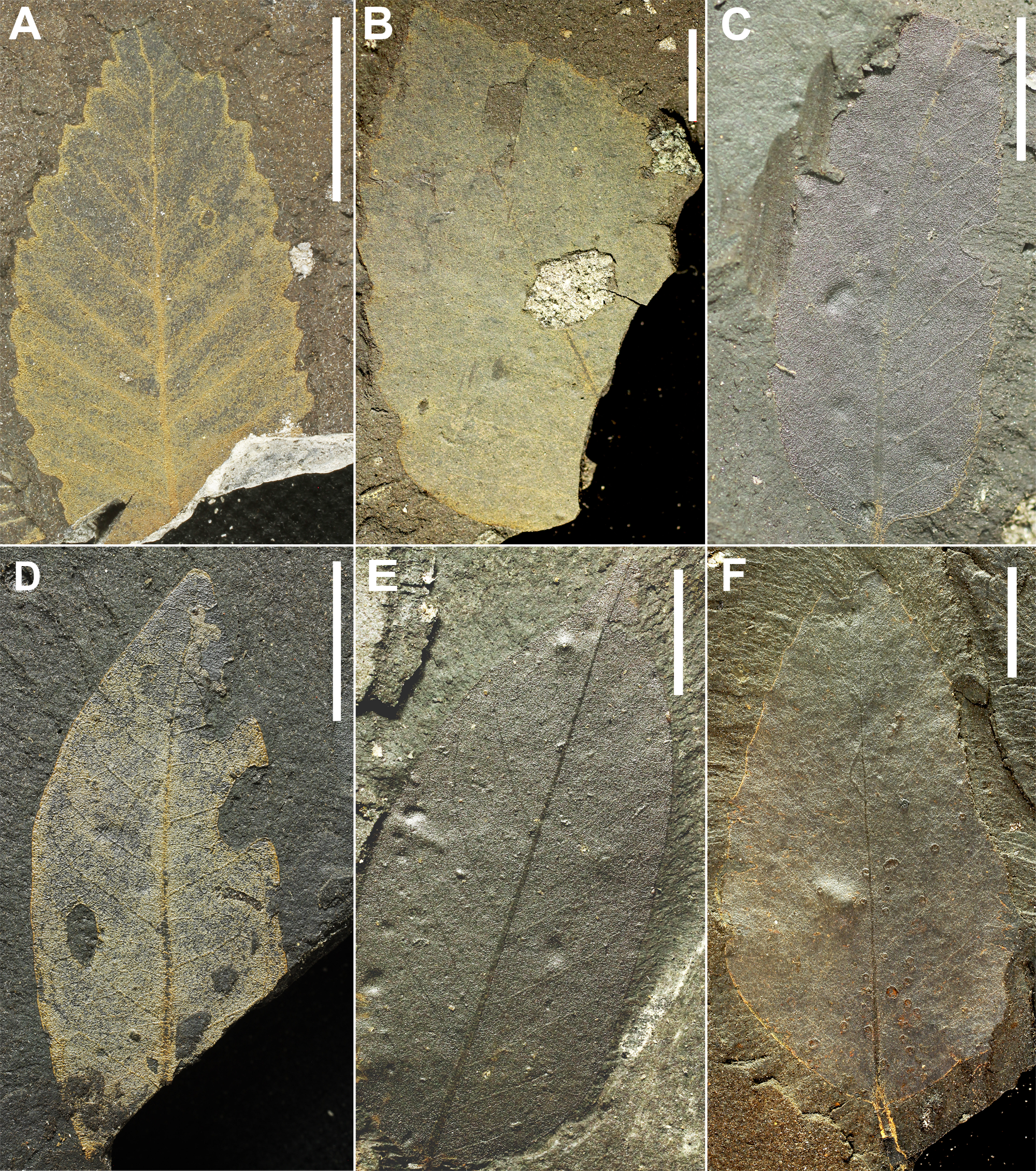

Supplement: Figure S1 — (A) Nothofagus 1; (B) Nothofagus 2; (C) indet. 7; (D) Lauraceae; (E) indet. 10; (F) Myrtaceae 1. Scale bars represent 10 mm. [file peerj-05-2985-s002.png]
